# Influence of age at entry and level of concentrate feeding on growth and carcass characteristics of feedlot-finished Tanzanian long-fat-tailed sheep

**DOI:** 10.1007/s11250-014-0570-0

**Published:** 2014-03-22

**Authors:** Eligy J. M. Shirima, Louis A. Mtenga, Abiliza E. Kimambo, Germana H. Laswai, Dyness M. Mgheni, Daniel E. Mushi, Dismas S. Shija, John G. Safari

**Affiliations:** 1 Department of Research, Training and Extension Services, Ministry of Livestock and Fisheries Development, P.O. Box 9152, Dar-es-Salaam, Tanzania; 2 Department of Animal Science and Production, Sokoine University of Agriculture, P.O. Box 3004, Morogoro, Tanzania; 3 Institute of Rural Development Planning, P.O. Box 138, Dodoma, Tanzania

**Keywords:** Concentrate, Feedlot, Growth, Slaughter characteristics, Long-fat-tailed sheep

## Abstract

A 4 × 3 factorial experiment was carried out to evaluate the effects of age at entry to feedlot (AEF) and levels of concentrate feeding (LCF) on body weight gain, feed utilization and killing out characteristics of Tanzanian long-fat-tailed castrate sheep. The AEF points were 9, 12, 15 and 18 months, designated as AEF9, AEF12, AEF15 and AEF18, and the LCF were 50, 75 and 100 % of ad libitum concentrate intake designated as LCF50, LCF75 and LCF100, the last representing ad libitum concentrate intake with 10 % refusal rate. Grass hay as basal diet was offered ad libitum to each sheep. Daily feed intake and weekly live weight were recorded for a period of 84 days. Animals were slaughtered and carcass and non-carcass parameters were recorded. Dry matter intake (DMI) of hay decreased while DMI of concentrate increased (*p* < 0.01) with increasing LCF. Daily gain in high level (LCF100) was 93.1 g/day, almost twofold higher than that in low level (LCF50) of feeding (39 g/day). Overall dressing percentage ranged from 40.7 to 46.5 % and increased with increasing AEF. The proportion of carcass bone decreased (*p* < 0.05) with increasing AEF while that of fat increased (*p* < 0.05) with increasing LCF. Age at entry × level of concentrate feeding interaction was detected for DMI, feed conversion ratio (FCR), slaughter body weight (SBW), muscle/bone ratio and bone (as % cold carcass weight (CCW)), but the effect was not regular. Entering fattening at 18th month seems too late, hence to get in the shortest time the highest output slaughter and carcass weights, fattening should start latest at 15 month.

## Introduction

In Tanzania, the three dominant indigenous sheep breeds are Tanzanian long-fat-tailed sheep, Blackhead Persian and Red Maasai, and they are all distributed mainly in semi-arid regions of the country. The Tanzanian long-fat-tailed sheep (TLS) are almost twofold much more numerous in Tanzania than the other two breeds and are well known for their stoutly built bodies and for good meat production (MLDF 2010). Compared with other indigenous breeds, the TLS have medium frame size bodies ranging from 25 to 40 kg at maturity (Devendra and McLeroy 1982); Devendra and McLeroy (1982) also pointed out that the long, thick tail condition of the TLS made them much more adaptable during the period of critical feed shortage. The Tanzanian long-fat-tailed sheep are important sources of meat and income in the farming system of agro-pastoralists despite their poor performance in terms of growth and meat production. The reason for the low productivity in these animals has been mainly associated with lack of sufficient good quality feeds. The sheep are raised extensively from natural pastures where they experience long periods of feed scarcity. In addition, the nutrients supplied from seasonal natural pastures are inadequate for maintenance and growth resulting in poor growth rates and prolonged periods to market slaughter weight of 25 kg (MLDF 2010). This type of management system might render sheep keeping less economical. One of the alternatives to address this problem is to develop a system of finishing sheep on high-energy supplementary feeds before sending them to the market. However, sheep growth performance, yield and quality of carcass and non-carcass parts under feedlot conditions may differ depending on the factors such as breed and age of the animal when entering feedlot or the duration of staying in the feedlot as well as the type and amount of diet used during fattening (Titi et al. 2008). The increase of feed offered in feedlot has been reported to increase growth performance and meat yield in sheep (Pasha 2006). It has been reported that after a certain age, growth rate of sheep is reduced making fattening uneconomical (Sultana et al. 2010). Increasing levels of concentrate feeding in adult sheep may lead to increased levels of carcass fatness, which in turn improves tenderness due to presence of intramuscular fats (Nishimura 2010). Decreasing AEF may result into low carcass weights and possibly more yield of non-carcass parts that are less valuable (van der Westhuizen 2010).

There is limited research information on the appropriate age at which TLS should enter into the finishing stage and on the level of feeding for improving growth and carcass characteristics. The present study was therefore intended to determine the effects of age at entry to the feedlot and levels of concentrate feeding on growth performance and carcass characteristics of Tanzanian long-fat-tailed sheep.

## Materials and methods

### Description of the study site

The feedlot trial was conducted at Kongwa Pasture Research Centre, and animals were slaughtered at Dodoma abattoir, both located in Dodoma region in Central Tanzania (6° 10′ 23″ S 35° 44′ 31″ E), at 1,120 m above sea level. The climate is semi-arid with average annual rainfall of 550 mm and minimum and maximum temperatures of 14 and 32 °C, respectively.

### Experimental design and treatments

One hundred and twenty castrated Tanzanian long-fat-tailed sheep aged 9, 12, 15 and 18 months with average initial weights of 14.2 ± 1.23, 17.9 ± 2.01, and 20.4 ± 2.15 and 25.1 ± 3.22 kg, respectively, were subjected to a 4 × 3 factorial design experiment. The age at entry to feedlot (AEF) of 9, 12, 15 and 18 months was designated as AEF9, AEF12, AEF15 and AEF18, and the three levels of concentrate feeding (LCF) were 50, 75 and 100 % of ad libitum concentrate offer, designated as LCF50, LCF75 and LCF100, the last representing ad libitum concentrate intake with 10 % refusal. Each AEF group composed of 30 animals, which were randomly assigned into the LCF, each with 10 animals per treatment.

### Source of experimental animals

Experimental animals were purchased from livestock keepers in Dodoma region, Tanzania. The age of sheep was estimated based on the incisor teeth stage according to the method developed by Owen et al. (1977) and records from farmers’ memory. The animals were identified by ear tagging and weighed on three consecutive days to obtain average initial body weight. The animals were injected with ivermectin (Kelamectin® 1 %, KELA N.V., 2320 Hoogstraten, Belgium) subcutaneously at a dose of 0.2 mg/kg body weight for treatment and control of endo- and ecto-parasites.

### Source of experimental feeds and feeding

*Cenchrus ciliaris* (buffel grass, African foxtail grass) hay was harvested at Kongwa Pasture Research Centre in May 2010. Ingredients for concentrate were liquid molasses purchased from Mtibwa Sugar Industry Company while the other ingredients were purchased from local agricultural input suppliers in Dodoma region. The grass hay was used as roughage while the concentrate diet was formulated from 66.3 % molasses, 15.5 % maize bran, 11.5 % cotton seed cake, 4.6 % rice polishing, 1.6 % urea, 0.4 % mineral and 0.1 % lime to contain 10.9 MJ metabolizable energy (ME)/kg dry matter (DM) and 160 g CP/kg DM. Fresh hay and concentrate were weighed and provided daily in the morning at 08.00 h in separate containers, and water was provided ad libitum. Dietary treatments were LCF50 and LCF75 where the amount of concentrate on offer consisted 50 and 75 %, respectively, of ad libitum concentrate intake (as fed basis). The third dietary treatment, LCF100, involved feeding concentrate ad libitum allowing 10 % refusal rate. The amount of concentrate offered to LCF50 and LCF75 groups was adjusted after every 7 days as the LCF100 group changed its intake. Dry matter intake (DMI) of feed offered and daily feed refusals were recorded every morning to derive daily feed intake. The amount of energy and protein fed per metabolic body weights was computed from the DMI of respective hay and concentrate rations using the metabolizable energy values of 6.4 and 12.8, respectively obtained from near-infrared reflectance spectrophotometer results in Table 1.

**Table 1:** Dry matter and chemical composition of experimental feeds

Item	Grass hay	Concentrate
Dry matter (g/kg)	893.0	830.6
Chemical composition (g/kg DM)		
Ash	57.0	88.7
Crude protein	47.8	160.5
Neutral detergent fibre	860.0	454.0
Acid detergent fibre	642.0	229.4
Metabolizable energy (MJ/kg DM)	6.39	12.8

### Experimental animals and measurements

The animals were housed and fed in a group of two animals per pen. An adaptation period of 14 days was allowed for sheep to get used to the experimental diet prior to the 84-day feedlot trial. Feed samples of hay and concentrate offered were collected every morning, bulked according to treatments, and then sub*-*sampled weekly to obtain representative samples. All animals were weighed and recorded weekly during the adaptation and experimental periods. The feeds and animals were weighed using spring balance (HANSON™ Model No. 21, H. Enterprises Bombay, India Max. 100 kg, calibrated at 5 kg with precision ±0.5 g). At the end of the growth trial, the animals were weighed on three consecutively days, and final body weight of each animal was obtained by averaging the weights. Average daily gain (ADG, g/day) was obtained by the difference between the final body weight (kg) and the initial body weight (kg) divided by the number of days in feedlot and multiplied by thousand. At the end of the feeding trial, feed and water were withheld overnight, and the animals were weighed to record the slaughter body weight.

### Slaughter procedures and measurements

The animals were slaughtered following standard procedures as described by Colomer-Rocher et al. (1987). Carcass which included the kidneys and pelvic fat was weighed within 6-h post-mortem and recorded as hot carcass weight (HCW). The carcasses were then split into left and right sides through the median plane using a handsaw. The split carcass halves were chilled at 4 °C overnight and then weighed and recorded as cold carcass weight (CCW). Non-carcass parts, which included the skin, head, hocks, full gastrointestinal tract (GIT), pluck (the heart, liver, lungs and trachea), tail fat and internal fats (fats from thoracic and intestines), were weighed and recorded. Full GIT was weighed and emptied, washed off its content and then re-weighed to obtain the empty GIT weight. Digestive content (GIT fill) was obtained by the difference in weight between the full GIT and empty GIT weight. GIT fill was then subtracted from the slaughter body weight (SBW) to determine empty body weight (EBW). Dressing percentage was calculated as the ratio of CCW/EBW times hundred.

### Carcass physical composition

The left hand sides of the carcasses were then jointed into seven wholesale cuts according to AUS-MEAT (1998) namely the neck, ribs, breast, loin, chump, hind leg and shoulder. Each cut was further dissected into components of lean meat, fat and bone and trimmings. The four components were weighed separately and then multiplied by 2 to determine their relative proportions to the cold carcass weight.

### Chemical composition of feed samples

The feed samples were analyzed for dry matter, ash, crude protein, neutral detergent fibre, acid detergent fibre and metabolizable energy using near-infrared reflectance spectrophotometer (NIRS) instrument (NIRSystems 5000 Firmware Version 156, USA). The instrument was specifically calibrated for hay and forage formula (mhaygpfe.eqa) and concentrate mixed rations formula (tmrgpfe.eqa) of NIRS procedure as described by Berglund et al. (1990) and Aufrere and Michalet-Doreau (1988), respectively.

### Statistical analysis

Statistical analysis was conducted for a 4 × 3 factorial experiment with four ages at entry and three levels of concentrate feeding as main effects. All data were analyzed as a completely randomized design with PROC MIXED of SAS (2001). Individual animal was used as the experimental unit in the model; therefore, residual error was used to test the main effects and interaction effects. Covariance analysis was done to correct for the effect of initial body weight within the entry ages for growth parameters, and final body weight were used for carcass composition data. Least square means were calculated for all measured variables, whereas the LSD test was used to determine significant differences (*p* < 0.05).

## Results

### Chemical composition of feeds

The DM content of hay and concentrate mixture was 893 and 830.6 g/kg DM, respectively (Table 1). The cell wall content in terms of acid detergent fibre of grass was 642 and 229.4 g/kg DM for concentrate diet. The metabolized energy content of 12.8 ME/kg DM for the mixed diets was higher than that of initially estimated values (10.6 ME/kg DM) from the literature during the planning of the experiment.

### Feed intake and growth performance

There was an interaction between AEF and LCF on the DMI of hay and concentrate diets (Table 2). The DMI as percentage of body weight ranged from 3.04 to 4.24 and was significantly higher (*p* < 0.01) in older (AEF18) than younger (AEF9) lambs, with AEF12 and AEF15 being intermediate. The overall intake as percentage of body weight increased (*p* < 0.01) as the LCF increased. LCF100 showed the highest (*p* < 0.05) intake as percentage body weight (BW) as well as the proportion of metabolic weight (BW^0.75^). The amount of feed required for a kilogram gain increased as the age at entry increased. More feed (14.3 kg) was required for each kilogram gain for the AEF18 castrates than AEF15 (8.07 kg), AEF12 (7.2 kg) and AEF9 (6.42 kg). There was also an interaction between AEF and LCF on feed conversion ratio (FCR). The daily gain increased with increasing LCF, with animals in LCF100 treatment showed the highest (*p* < 0.05) gain (93.1 g/day) which was 2.4- and 1.3-folds higher than that in LCF50 and LCF75 groups, respectively.

**Table 2:** Least square means ± SE for feed intake and growth performance of lambs under different ages at entry to feedlot (AEF) and levels of concentrate feeding (LCF) (*n* = 120)

Traits	AEF (months)					LCF (%)				*p* value		
	9	12	15	18	SE	50	75	100	SE	AEF	LCF	AEF × LCF
Intake (g DMI/day)												
Hay	217.7	220.6	217.6	209.1	2.30	247.1^a^	210.0^b^	191.1^c^	2.00	NS	**	**
Concentrate	501.6^d^	645.0^c^	698.9^b^	885.1^a^	8.60	487.3^c^	701.1^b^	859.7^a^	4.90	**	**	**
Total	719.3^d^	865.6^c^	916.5^b^	1,094.3^a^	9.27	734.4^c^	911.0^b^	1,051.4^a^	5.30	**	**	**
Intake (% BW)	3.04^c^	3.48^b^	3.57^b^	4.24^a^	0.10	3.26^c^	3.62^b^	3.86^a^	0.04	**	**	NS
Intake (g/kg W^−75^/day)	33.0^d^	36.3^c^	38.1^b^	45.6^a^	0.74	31.6^c^	38.9^b^	44.3^a^	0.42	**	**	NS
Energy intake (MJ/kg W^−0.75^/day)	6.44^d^	6.68^c^	6.74^b^	6.82^a^	6.50^c^	6.72^b^	6.82^a^	8.42^a^	0.01	**	**	NS
Protein intake (g/kg W^−0.75^/day)	63.0^d^	65.2^c^	65.8^b^	67.0^a^	0.01	63.8^c^	65.4^b^	66.6^a^	0.01	**	**	NS
Daily gain (g/day)	69.8	69.7	66.1	66.5	3.91	39.0^c^	71.9^b^	93.1^a^	3.38	NS	**	NS
FCR (kg DMI/kg gain)	6.42^c^	7.24^c^	8.07^b^	14.3^a^	1.05	11.6^a^	7.81^b^	7.65^b^	0.91	**	**	*

*SE* standard error of mean, *DMI* dry matter intake, *BW* body weight, *FCR* feed conversion ratio, *NS* not significant

*p < 0.05; ** *p* < 0.01

^abcd^Least squares means with a common superscript in the same row are not significantly different (*p* > 0.05)

### Killing out characteristics

Total body weight gain differs (*p* < 0.05) with increasing LCF, whereby an increase of 2.76 kg (84.1 %) was observed when dietary levels changed from LCF50 to LCF75 and only 0.78 kg (29.5 %) when the levels changed from LCF75 to LCF100 (Table 3).

**Table 3:** Least square means ± SE for slaughter characteristics of lambs under different ages at entry to feedlot (AEF) and levels of concentrate feeding (LCF) (*n* = 120)

Traits	AEF (months)					LCF (%)				*p* value		
	9	12	15	18	SE	50	75	100	SE	AEF	LCF	AEF × LCF
Initial body weight (kg)	13.9^d^	18.2^c^	20.9^b^	25.8^a^	0.47	19.3	19.5	20.5	0.40	**	NS	NS
Final body weight (kg)	19.9^d^	23.9^c^	26.3^b^	31.2^a^	0.51	22.6^c^	25.3^b^	28.3^a^	0.46	**	**	NS
Slaughter body weight (kg)	18.8^d^	22.8^c^	24.8^b^	29.5^a^	0.50	21.1^c^	23.8^b^	27.0^a^	0.44	**	**	*
Total body weight gain (kg)	5.86	5.85	5.55	5.59	0.33	3.28^c^	6.04^b^	7.82^a^	0.28	NS	**	NS
EBW (kg)	17.6^d^	21.7^c^	23.6^b^	28.6^a^	0.50	20.1^c^	22.6^b^	26.0^a^	0.40	**	**	NS
EBW (as % SBW)	93.6^c^	95.2^b^	95.2^b^	97.0^a^	0.61	95.7^b^	95.0^b^	96.7^a^	0.52	**	**	NS
HCW (kg)	7.87^d^	10.3^c^	11.0^b^	13.3^a^	0.28	8.88^c^	10.2^b^	12.0^a^	0.23	**	**	NS
HCW (as % SBW)	41.7^c^	43.1^b,c^	44.5^a,b^	45.1^a^	0.55	42.7^b^	43.3^b^	44.7^a^	0.22	**	*	NS
Cold carcass weight (kg)	7.69^d^	9.69^c^	10.9^b^	12.2^a^	0.27	8.62^c^	10.0^b^	11.4^a^	0.20	**	**	NS
CCW (as % SBW)	40.7^c^	42.5^b^	44.0^a^	44.7^a^	0.54	41.8^b^	42.8^b^	44.3^a^	0.19	**	*	NS
Gut fill (kg)	2.26^b^	2.21^b^	2.68^a^	2.61^a^	0.11	2.51	2.48	2.34	0.20	**	NS	NS
Gut fill % SBW	12.0^a^	9.68^b^	10.8^b^	8.84^c^	0.45	11.9^a^	10.4^b^	8.66^c^	0.47	**	**	NS
Dressing percentage	44.6^b^	45.1^a,b^	46.5^a^	46.4^a^	0.55	44.8^b^	45.1^a,b^	46.4^a^	0.50	**	**	NS

*SE* standard error of mean, *SBW* slaughter body weight, *EBW* empty body weight, *HCW* hot carcass weight, *NS* not significant

*p < 0.05; **p < 0.01

^abcd^Least squares means (LSM) with a common superscript in the same row are not significantly different (*p* > 0.05)

A quantitative interaction (*p* < 0.05) between the AEF and LCF was observed for SBW. Hot and cold carcass weights increased with increasing AEF. For hot carcass weight, the *r* ^2^ was 0.786 (*p* < 0.01). The proportion of gut fill (as % SBW) decreased (*p* < 0.01) with increasing both AEF and LCF. The dressing percentage (on EBW basis) of AEF12, AEF15 and AEF18 lambs ranged between 45.1 and 46.5 % and were about 2 % higher compared with those from AEF9 lambs. The proportion of non-carcass parts (as % SBW) decreased (*p* < 0.05) with increasing both AEF and LCF (Table 4).

**Table 4:** Least squares means ± SE for non-carcass components (NCC) of castrated Tanzanian long-fat-tailed sheep under different ages at entry to feedlot (AEF) and levels of concentrate feeding (LCF) (*n* = 120)

Variable	AEF (months)					LCF (%)				*P* value		
	9	12	15	18	SE	50	75	100	SE	AEF	LCF	AEF × LCF
NCC weight (kg)												
Pluck	0.81^c^	0.98^b^	1.04^b^	1.16^a^	0.03	0.91^b^	1.01^b^	1.01^a^	0.02	**	**	NS
Kidneys	0.08	0.07	0.07	0.09	0.01	0.07^b^	0.08^a,b^	0.09^a^	0.01	NS	*	NS
GIT tract (full)	4.26^a^	4.55^b^	5.09^a^	5.26^a^	0.12	4.66	4.82	4.89	0.35	**	NS	NS
GIT tract (empty)	2.00^c^	2.34^b^	2.41^b^	2.65^a^	0.03	2.15	2.35	2.55	0.14	**	NS	NS
Head, skin and Hocks	3.87^c^	4.58^b^	4.86^b^	5.76^a^	0.11	4.30^c^	4.83^b^	5.16^a^	0.09	**	**	NS
Tail fat	0.88^c^	1.03^b,c^	1.17^b^	1.31^a^	0.42	1.00^b^	1.09^b^	1.20^a^	0.06	*	*	NS
NCC (as % SBW)												
Pluck	4.31^a^	4.37^a^	4.21^a,b^	3.96^b^	0.10	4.35^a^	4.28^a^	4.02^b^	0.08	*	*	NS
Kidneys	0.43^a^	0.32^b^	0.31^b^	0.31^b^	0.04	0.33	0.34	0.36	0.03	*	NS	NS
GI tract (full)	22.7^a^	20.0^b^	20.5^b^	17.8^c^	0.41	22.1^a^	20.3^b^	18.1^c^	0.36	**	**	NS
GI tract (empty)	10.7^a^	10.3^b^	9.72^c^	8.99^d^	0.19	10.2^a^	9.85^b^	9.45^c^	0.20	**	*	NS
Head, skin and Hocks	20.6	20.1	19.6	19.5	0.36	20.4^a^	20.3^a^	19.1^b^	0.31	NS	**	NS
Tail fat	4.70	4.53	4.71	4.43	0.41	4.74	4.58	4.46	0.36	NS	NS	NS

*SE* standard error of mean, *GIT* gastrointestinal tract, *SBW* slaughter body weight, *NS* not significant

*p < 0.05; **p < 0.01

^abcd^Least squares means with a common letter superscript in the same row are not significantly different (*p* > 0.05)

### Carcass composition

The weights of dissectible muscle, fat and bone were all increased (*p* < 0.01) with increasing AEF (Table 5). When expressed as a proportion of total cold carcass weight, the percentage of carcass muscle and fat were not remarkably affected by AEF while we cannot pronounce on the effect on bone per cent due to the interaction between AEF and LCF. An interaction of AEF and LCF was also seen on the ratio of muscle/bone in the carcass. There was also a decrease (*p* < 0.05) in muscle/fat ratio by almost 35.8 % as the concentrate level increased from LCF50 to LCF100.

**Table 5:** Least squares means ± SE for carcass tissues from Tanzanian long-fat-tailed lambs under different ages at entry to feedlot (AEF) and levels of concentrate feeding (LCF) (*n* = 120)

Traits	AEF (months)					LCF (%)				*P* value		
	9	12	15	18	SE	50	75	100	SE	AEF	LCF	AEF × LCF
Tissue (kg)												
Muscle	4.39^d^	5.62^c^	6.19^b^	7.50^a^	0.17	5.25^c^	6.03^b^	6.49^a^	0.15	**	**	NS
Fat	1.67^c^	2.06^b,c^	2.36^b^	3.30^a^	0.20	1.67^c^	2.37^b^	3.01^a^	0.17	**	**	NS
Bone	2.05^c^	2.41^b^	2.60^a,b^	2.79^a^	0.10	2.31	2.50	2.58	0.08	**	NS	NS
Trimmings	0.19	0.17	0.23	0.27	0.04	0.25	0.21	0.17	0.04	NS	NS	NS
Tissue (% CCW)												
Muscle	53.3^b^	55.1^a^	54.6^a^	54.6^a^	1.20	55.7^a^	54.5^b^	52.9^c^	1.01	*	**	NS
Fat	19.8	19.4	20.4	23.1	1.30	17.1^c^	20.8^b^	24.2^a^	1.12	NS	**	NS
Bone	25.2^a^	23.8^a,b^	23.0^b^	20.4^c^	0.60	25.1^a^	22.8^b^	21.4^b^	0.50	**	**	*
Trimmings	2.59	1.63	1.99	1.92	0.54	2.77	1.88	1.45	0.47	NS	NS	NS
Muscle/fat ratio	3.01	3.36	2.97	2.66	0.35	3.60^a^	3.09^b^	2.31^c^	0.31	NS	*	NS
Muscle/bone ratio	2.13^b^	2.34^b^	2.41^b^	2.82^a^	0.11	2.25^b^	2.43^a,b^	2.59^a^	0.10	**	*	*

*SE* standard error of mean, *NS* not significant

*p < 0.05; **p < 0.01

^abcd^Least squares means with a common letter superscript in the same row are not significantly different (*p* > 0.05)

## Discussion

### Feed intake and growth performance

The higher feed intake of AEF18 lambs (as % BW) in the present study could be associated with both the age of the animals and the level of concentrate feeding offered, although the trend was not regular. As reported by AFRC (1993), feed intake of similar diets by animals is normally considered to be a function of BW or metabolic weight. In the present study, the feed intake ranged between 3.00 and 4.24 1 % of BW, and these values are slightly above to those reported by Safari et al. (2011) of 2.89 % in castrated Red Maasai sheep fed urea-treated straw and hay. The discrepancy to these findings might be attributed to difference in diets used and body weights of the animals (Mahgoub et al. 2000). These results are in consistent with those of Hagos and Melaku (2009) who reported higher feed efficiency in Afar rams fed concentrate mix than those fed tef straw roughage in Ethiopia. These findings are further supported by Mahgoub et al. (2000) who reported relatively high body weight gains and carcass composition through increasing the energy levels in the diets of growing Omani lambs. Castrates in AEF18 consumed the highest energy and protein (BW^0.75^) compared with animals in other age group. The increase of intake with increasing age at entry in the current findings is contrary to that observed by Sultana et al. (2010) on castrated native sheep in Bangladesh, who observed a decline in intake as the age of sheep increased when fed urea molasses based concentrate diets. The difference between these two observations might be due to differences in breed type of sheep, AEF and concentration of energy in the diet used.

The higher body weight gain for lambs on LCF100 compared with that on LCF50 can be attributed to increased availability of energy and protein from the high offer. Similarly, the ration of high energy (LCF100) offered allows more microbial population growth and therefore more basal ration (roughage) digestion and ultimately more provision of protein and energy to the animals (Safari et al. 2011). The growth rate (93.1 g/day) of lambs fed LCF100 was higher than that reported for Red Maasai sheep fed treated straw with hay (47.8 g/day; Safari et al. 2011) or castrated native sheep in Bangladesh (54.6 g/day; Sultana et al. 2010) for lambs of the similar age. However, these findings are lower than those reported in Katahdin lambs fed alfalfa hay (131 g/day; Wildeus et al. 2007) and far below the values reported for Dorset lambs fed different concentrate levels (449 g/day; Jacques et al. 2011). The observed differences in the daily gain could be caused by the differences in breed of the animals, AEF, efficiency of feed conversion and energy density consumed from the diet.

### Killing out characteristics

The heavier carcasses in LCF100 lambs were probably caused by their superior gains from higher intake of both energy and protein from the concentrate diet. The decrease proportion of gut fill to slaughter weight with increasing AEF is caused by the age and diet taken by the experimental animals. The proportion of gut fill is known to decrease with feeding low-roughage diets, and here, low intake of hay roughages and high intake of concentrate diets in animals fed LCF100 could be implicated. These findings are in agreement with those reported by Cañeque et al. (2003) who suggested that animals fed more concentrate-based diets have less developed digestive tract because their DMI contains less roughage. The observed hot carcass weight of older lambs (AEF15 and AEF18) is probably attributable to increased fatness in old aged animals. The carcass weights in AEF12 (10.3 kg) lambs and those in AEF15 (11.0 kg) and AEF18 (13.3 kg) lambs in the current study are almost similar to those reported from Assaf and Merino × Assaf sheep breeds (Rodríguez et al. 2011) and lower than those reported from other sheep breeds (14–21 kg) of comparable slaughter weights in the tropics (Jacques et al. 2011) The differences in the carcass weights among the AEF groups could be caused by the differences in their breed, age and initial weight at entry to feedlot, slaughter age, type of feed and carcass fatness level. The total body weight gain in LCF50, LCF75 and LCF100 groups might be profitable to producers if a full economic analysis is done as the carcasses above 10 kg are the ones that fetch higher premium price in most of the export markets in Tanzania (MLDF 2010).

The observed dressing out percentages (44.5 to 46.5 %) are slightly below the values reported (46–47 %) for other sheep breeds in the tropics (Rodríguez et al. 2011). These values are also lower than most of the dressing percentages reported from the tropical sheep breeds by other authors (Sen et al. 2011 and Abdullah and Qudsieh 2008) The differences could arise from breed, type of diet, age of the animals, and degree of fatness in the animal and slaughter weight. The lower dressing percentage among the AEF groups could also be caused by the differences in age of the experimental animals which were subjectively estimated by the researcher through the stage of the incisor teeth together with the farmer’s memory. This anomaly is possible because farmers in rural areas of Tanzania do not keep records of their animals’ ages. Other researchers working with various tropical sheep breeds (Abdullah and Qudsieh 2008) have also reported the association of dressing per cent and the degree of fatness and reduced gut fill. The decrease in non-carcass parts as proportion of SBW with increasing AEF in the current study was very much linked to the slaughter age and level of carcass fatness. Also, the higher proportion of the empty GIT in the AEF9 lambs could be attributed to their lower level of body fatness as compared with that in the AEF12, AEF15 and AEF18 groups. The current findings are consistent with those results by Hagos and Melaku (2009) that showed a decrease in weight of empty GIT (as percentage slaughter weight) with increase in concentrate intake with subsequent decrease in roughage intake. Similarly, Suliman and Babiker (2007) reported higher roughage intake stimulated GIT development that supported peristaltic movements involved in digestion. The decrease of proportion (as % slaughter weight) of GIT, head and hocks as the AEF increased is probably caused by their nature of development for being early maturing components; hence, they are relatively less affected by the dietary treatments applied in mature animals (Lawrie 1998). The decrease in proportion of non-carcass parts (as % SBW) as the AEF increased can be seen as a positive attribute for producers as more nutrients obtained from diet is used for the growth of the higher priced edible tissues (lean and fat).

### Carcass composition

The observed higher weights of carcass muscles, fat and bone tissues in favour of AEF18 animals compared with those of AEF9, AEF12 and AEF15 groups are chiefly due to heavier carcasses in the former than in the latter. The observed proportions to cold carcass weight (53.3–55.1 %) of muscle content are closer to 54 to 58 %, reported by Legesse et al. (2005) in most of the tropical sheep breeds and their crosses. These results are consistent with those observed in other comparative trials in Merino and Dorper sheep (van der Westhuizen 2010). In addition, the decrease of muscle and bone and increase of fat proportion (as % CCW) with increase of dietary levels is associated with the age of the animals and stage of growth in the lambs used. It has been reported by Lambuth et al. (1970) that during the growth of lambs, fat deposition will increase when the plateau phase of the growth curve is reached. In this case, AEF9 and AEF12 animals were considered to be younger than AEF15 and AEF18; therefore, they had relatively higher proportion of bone than fat deposition because the energy consumed was mainly used for growth, while with matured animals, excessive energy results to fat deposition.

## Conclusion

It is concluded that entering fattening at 18th month seems too late. To get in the shortest time the highest output weight, fattening should start at 15th month. Similarly, the most advantageous diet might be LCF75, but this should be confirmed by a cost-benefit analysis.
